# Monitoring SARS-CoV-2 variants in wastewater during periods of low clinical case surveillance in Ethiopia

**DOI:** 10.1128/msphere.00229-25

**Published:** 2025-07-29

**Authors:** Gebremedhin Gebremicael, Daniel Abera Dinssa, Atsbeha Gebreegziabxier, Yohannes Mengistu, Melak Getu, Dinknesh Chalchisa, Girma Berhanu, Firehiwot Mulugeta, Daniel Melese, Ashley Norberg, Sarah Snyder, Rajiha Abubeker, Saro Abdela, Abebaw Kebede, Abraham Ali, Sofonias K. Tessema, Tobias F. Rinke de Wit, Gemechu Tadesse, Yenew Kebede, Mesay Hailu, Masresha Tessema, Noah C. Hull, Getachew Tollera, Dawit Wolday

**Affiliations:** 1 Infectious diseases Research Directorate, Ethiopian Public Health Institute (EPHI) 128164, Addis Ababa, Ethiopia; 2 Nutrition, Environmental Health and Non-communicable diseases research directorate, EPHI, Addis Ababa, Ethiopia; 3 Department of Biochemistry and Biomedical Sciences, University of Dundee 3042, Dundee, Scotland, United Kingdom; 4 The Association of Public Health Laboratories (APHL Ethiopia), Addis Ababa, Ethiopia; 5 The Association of Public Health Laboratories (APHL), Bethesda, Maryland, USA; 6 Africa Centres for Disease Control and Prevention (Africa CDC), Surveillance and Disease Intelligence Division, Addis Ababa, Ethiopia; 7 Amsterdam Institute of Global Health and Development, Department of Global Health, Amsterdam University Medical Center, Amsterdam, the Netherlands; 8 Department of Biochemistry and Biomedical Sciences, Michael G. DeGroote Institute for Infectious Diseases Research and McMaster Immunology Research Center, Faculty of Health Sciences, McMaster University 3710, Hamilton, Ontario, Canada; Instituto de Biotecnologia/UNAM, Cuernavaca, Morelos, Mexico

**Keywords:** wastewater based epidemiology, SARS-CoV-2, COVID-19, genomic surveillance, Freyja analysis

## Abstract

Wastewater-based genomic surveillance is a cost-effective approach for tracking outbreaks like severe acute respiratory syndrome coronavirus 2 (SARS-CoV-2). A 2023 study in Addis Ababa, Ethiopia, revealed two infection waves in March and August, with the latter undetected by clinical surveillance. This study analyzed the viral spread and evolution in the population during periods of low reported cases. Viral concentration was performed following the Ceres Technology protocol, and RNA was extracted using the QIAamp Viral RNA Mini Kit. Quantitative PCR was performed using the TaqPath COVID-19 Kit. Samples with cycle threshold values ≤32 were used for sequencing. Library preparation and sequencing were performed using the Illumina COVIDSeq protocol, and data analysis was conducted using the Freyja pipeline on Terra.bio. SARS-CoV-2 viral load in wastewater began rising on 6 March 2023, peaking on 16 March 2023, before declining until early May 2023. A resurgence occurred from 3 to 21 August 2023. In March 2023, XBB.1.5 (34%), XBB* (20%), and CH.1.1 (15%) were dominant. By April–May 2023, XBB.1.5 rose to 51% but declined to 14% in June 2023, while XBB* increased to 41%. In August 2023, XBB* (52%) and XBB.1.5 (31%) co-dominated. Key spike protein mutations (G142D, V213G, T478K, S494P, S477N) correlated with higher viral loads. Wastewater surveillance of SARS-CoV-2 reveals seasonal and behavioral transmission patterns. A March peak linked to XBB.1.5 and XBB* saw XBB.1.5 dominance through May 2023, later declining. An August 2023 resurgence with XBB* co-dominance suggests viral evolution and waning immunity. Key spike mutations correlate with higher viral loads, emphasizing wastewater surveillance’s predictive value.

**IMPORTANCE:**

This study highlights the critical role of wastewater monitoring in detecting and tracking severe acute respiratory syndrome coronavirus 2 (SARS-CoV-2) outbreaks, particularly in regions with limited clinical reporting. Using genomic analysis tools like Freyja enables the detection and monitoring of SARS-CoV-2 variants by untangling mixed signals to track viral evolution and mutations. This unbiased method offers a comprehensive assessment of virus prevalence, including asymptomatic cases, making it a key supplement to clinical surveillance. By addressing gaps and biases in testing, the detection of two distinct viral waves in Addis Ababa, including one missed by patient-based surveillance, underscores the effectiveness of this approach. The shifting dominance of Omicron sub-lineages, such as XBB.1.5 and XBB*, and their spike protein mutations provide essential insights into viral evolution and transmission dynamics. The connection between specific mutations and increased viral loads further suggests potential impacts on viral fitness and transmissibility. These results reinforce the need to integrate wastewater surveillance into public health strategies to support clinical surveillance, enable early detection of emerging variants, and support timely interventions. Moreover, wastewater surveillance can be extended to monitor other pathogens and antimicrobial resistance, making it an essential tool for pandemic preparedness and ongoing public health management.

## INTRODUCTION

Genomic surveillance provides a powerful approach to monitoring known or unknown pathogens and analyzing their genetic similarities and differences (1). Sequencing of infected patient case samples is necessary for clinical monitoring of the coronavirus disease 2019 (COVID-19) pandemic, but it doesn’t capture community transmission of COVID-19 in a rapid, cost-effective, and unbiased manner, especially during periods of low clinical case surveillance (2). Moreover, clinical genomic surveillance is focused on monitoring patients who exhibit symptoms and who are a biased representation of the population (3). Current data suggest that 35%–45% of all severe acute respiratory syndrome coronavirus 2 (SARS-CoV-2) infections account for asymptomatic cases worldwide (4–6). The percentage of such cases in Africa and Ethiopia is even higher, reaching 90% (7). Nevertheless, asymptomatic individuals have the potential to transmit the virus quickly because they may not seek medical treatment or take preventative action to lower the risk of transmission (8, 9). In addition, single-patient clinical genomic surveillance requires relatively high viral loads in the sample with a Ct value less than 30 (10). Considering all this, a patient-based picture of SARS-CoV-2 viral diversity and transmission is biased and only partly represents what is happening at the population level.

A more representative measure of COVID-19 epidemic dynamics at the population level is wastewater surveillance, since both symptomatic and asymptomatic individuals shed SARS-CoV-2 in their urine and feces (11–13). Wastewater-based surveillance of epidemics has long been used in public health, especially in the Global Polio Eradication Initiative (14). During COVID-19, there was an exponential increase in wastewater-based genomic surveillance and its use as a tool for decision-making and for setting priorities for mitigating the pandemic (15). As part of an early warning system to battle COVID-19, the U.S. Centers for Disease Control and Prevention created the National Wastewater Surveillance System with a focus on SARS-CoV-2.

The wastewater-based approach has been successful in globally tracking COVID-19 variants (16–18). However, in Africa, very few countries used this methodology consistently (19, 20). South Africa proved most experienced in wastewater genomic surveillance, even predicting upcoming lineage transitions (21). Some work was also done in Malawi (20).

Ethiopia observed a decline in COVID-19 cases since the end of 2022 (22). In our previous study, we indicated that this can be partly explained by decreasing trends in SARS-CoV-2 testing (23). A single wastewater sample can reflect the infection status of entire communities, eliminating the need for individual testing and enabling broad, efficient surveillance (2). By applying whole genome sequencing, this approach consolidates data collection and supports timely interventions that can prevent widespread transmission and reduce the costs associated with large-scale outbreaks. Wastewater monitoring enables early outbreak detection by identifying pathogens like SARS-CoV-2, mPox, and Ebola days to weeks before clinical cases appear. Continuous sampling supports trend analysis over time, providing early warnings of rising or declining infection rates signaling the emergence of infectious diseases. In this study, we also indicated that wastewater-based estimation in Addis Ababa is significantly higher than reported cases. The dominant variant or lineage of SARS-CoV-2 during 2022 in Ethiopia was Omicron BA.1 and BA.4/5 (24). For the year 2023, there was little individual clinical sequencing data available, due to the decreasing alarm around COVID-19, a consequence of a decrease in testing appetite and potentially an additional decrease in local sequencing capacity due to cessation of COVID-19 reagents delivery by international aid efforts. A new era of COVID-19 had started, where information on the epidemic had to be collected in a different, more cost-effective, and representative manner. This study presents the SARS-CoV-2 information obtained through wastewater sequencing during two COVID-19 waves in March and November 2023 in Addis Ababa, Ethiopia.

## RESULTS

A total of 95 wastewater samples with Ct value ≤32 underwent sequencing and the amplification process. Of the total, 79 (80%) of the samples passed the sequence quality threshold and had over 25 read length, making them eligible for both the variant and lineage analysis (Table S1). In total, 59 (75%) of the 79 samples had >2× sequencing coverage of the whole genome; these samples were utilized in the Freyja_Plot analysis and Freyja_Dashboard visualization.

### Detection of SARS-CoV-2 Omicron lineage from wastewater samples and clinical samples

**Fig 1:**
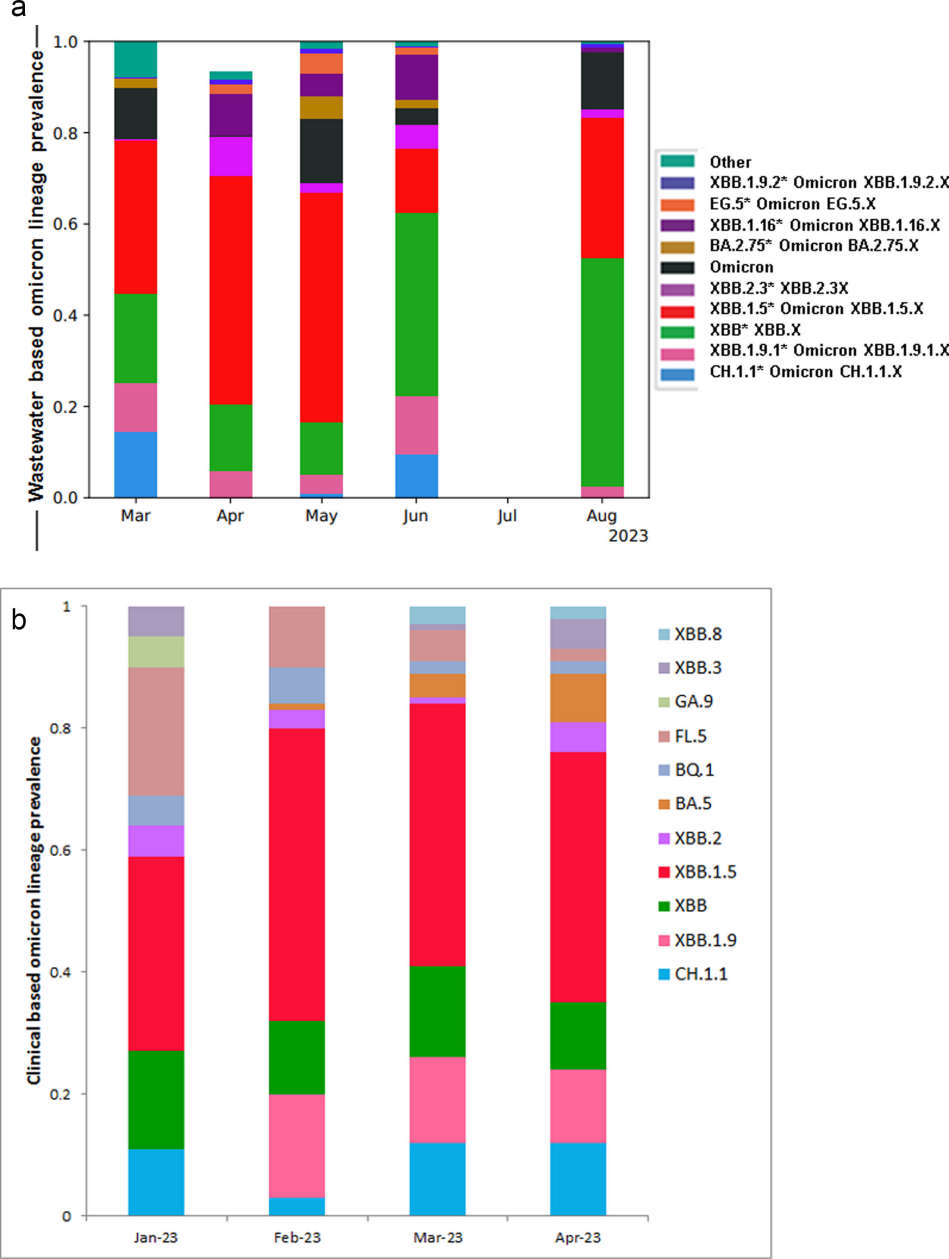
Trend of Omicron lineage prevalence. (a) Wastewater Omicron lineage prevalence. (b) Clinical-based Omicron lineage prevalence.

The frequency of several SARS-CoV-2 lineages in wastewater across time, from March to August 2023, is illustrated in Fig. 1a. All of the samples collected in June failed due to low-quality fastq reads, due to low viral load. In March 2023, XBB.1.5 was the dominant variant at 34% prevalence. The second and third dominant variants were XBB* (20%) and CH.1.1 (15%), while other variants like Omicron, XBB.1.9.2, and BA.2.75 were present at lower levels, each around 10% or less. The XBB.1.5 variant prevalence increased to 51% in April and May 2023. XBB.1.16 (9%) and XBB.2.3 (8%) were detected in April 2023; EG.5, XBB*, and XBB.1.9.2 remained less than 10%. In June 2023, the XBB.1.5 variant’s prevalence sharply decreased to 14%, while XBB* increased to 41%, thus becoming dominant. The prevalence of XBB.1.9.1, CH.1.1, and XBB.1.16 increased slightly in June 2023, while BA.2.75, Omicron, and EG.5 slightly decreased. In August 2023, the XBB* and XBB.1.5 variants increased to 52% and 31%, respectively, thus becoming co-dominant variants. XBB.1.9.1, XBB.1.16, XBB.2.3, and XBB.1.9.2 remained low (<10%) during August 2023.

We did not find Ethiopian clinical SARS-CoV-2 sequencing data in NCBI or other public databases since May 2023. However, we could compare clinical and wastewater data sequenced in March and April 2023 (Fig. 1b). Both identified XBB.1.5 as the dominant lineage during this 2-month study period in Addis Ababa. Additionally, we observed a significant patient prevalence of XBB*, CH.1.1, FL.2, and BA.5 lineages, consistent with wastewater detection results. Low levels of XBB.8, XBB.1.9, BQ.1, and other Omicron lineages were also detected in clinically sequenced samples during this period.

### Circulating Omicron lineages correlated with the trend of viral concentration in wastewater

**Fig 2:**
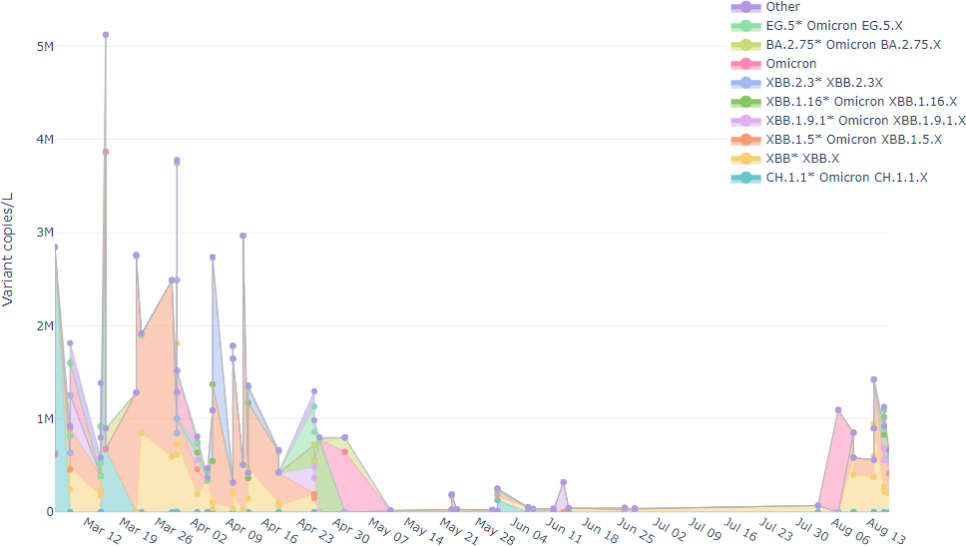
Trend of SARS-CoV-2 Omicron lineages’ viral loads in the wastewater of Addis Ababa. Viral load of each omicron lineage at each collection date throughout the year.

In clinical case reporting, there was only one Omicron wave during this study, but our studies in wastewater indicated two waves (24). The correlation of the viral load of the ORF1ab target gene and circulating Omicron lineages of those samples that passed sequence quality (59 wastewater samples) and had 2× coverage is presented in Fig. 2.

**Fig 3:**
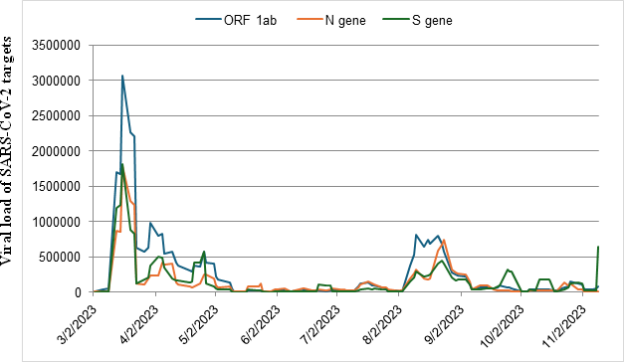
Trend of cumulative genome copies of SARS-CoV-2 gene targets in wastewater treatment plants. The data represent the average number of SARS-CoV-2 gene copies for the ORF-1ab gene, N gene, and S million genome copies per L-day of wastewater sample obtained in the untreated wastewater samples from the wastewater treatment plants.

On 6 March 2023, SARS-CoV-2 viral load in wastewater began to increase, reaching a peak on 16 March 2023 (Fig. 3). Then the viral load started to decline until the beginning of May 2023. The most common Omicron lineages detected during the March 2023 peak were CH.1.1 (76%) and BA.2.75 (24%). Subsequently, the XBB.1.5 and XBB* variants became dominant from March 22 to the end of April 2023. The viral load in the wastewater remained relatively constant from 11 May to 3 August 2023, with the XBB* variant being most prevalent. Subsequently, there was a sharp increase in viral load from 3 to 21 August 2023. Until 10 August 2023, the XBB* variant remained the most dominant. Then, as of 14 August 2023, a second Omicron wave emerged, with XBB.1.5 and XBB.1.16 becoming co-dominant alongside XBB*.

### Amino acid mutational analysis

**Fig 4:**
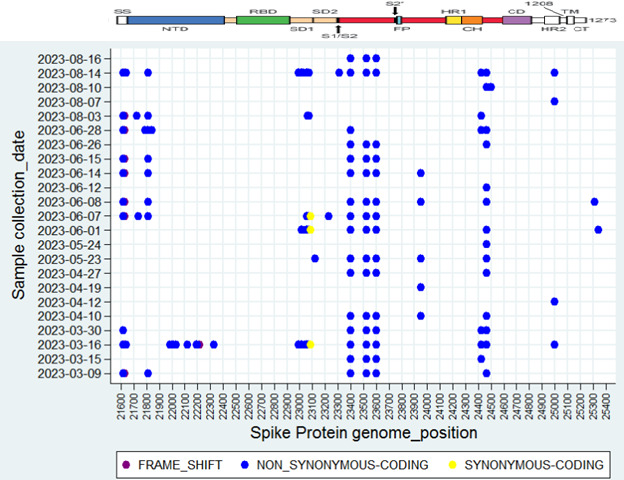
The amino acid mutational analysis of the SARS-CoV-2 scatter plot. The dots in the figure represent the amino acid mutation at each collection date.

In the ORF1a and ORF1b regions of SARS-CoV-2 of the overall study samples, 57 and 23 amino acid mutations (substitutions and/or deletions) were observed, respectively (Fig. 4; Table S2). Additionally, amino acid mutations were observed in various SARS-CoV-2 proteins, including ORF3a (L3C, S74F, A98S, A103V, G196R, G224S, M260, P240L, T223I), Envelope (E) (T9I, T11A), Membrane (M) (A63T), ORF6 (D61H, D61V), ORF7a (I3F, P34L, E41*, S60C, Q62*, Q62H, Y97H, I103V, K119N), ORF7b (L14G, A43P, A43S, A43V), ORF8 (G8*, P36L, L60*, V62L, E110*, Y111C, F120L, F120V, I121L, I121S), and Nucleocapsid (N) (P13L, T16M, E31del, R32del, S33del, S33G, A50V, R88L, P151S, A155V, S180I, A125S, R203K, G204R, A267T, H300Y, I357L, S412G, S413R) (Fig. 4).

**Fig 5:**
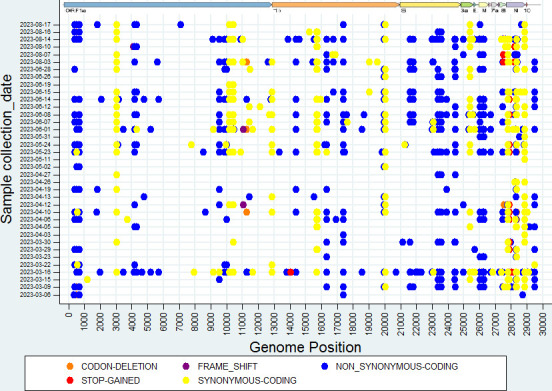
The amino acid mutational analysis of the SARS-CoV-2 spike protein scatter plot. The dots in the figure represent the amino acid mutation at each collection date.

The spike gene exhibited noteworthy signature mutations, including T19I, A26S, L54F, F59L, V83A, T95A, G142D, K147E, W152R, F157L, K187T, V213G, I210V, S221R, and G257S in the N-terminal domain (NTD); S477N, T478K, T478R, E484A, F486L, F486S, F490S, S494P, Q498R, N501Y, Y505H, and P521T in the receptor binding domain (RBD); N556S, T583E, D614G, H655Y, N679K, P681H, and D796Y in S2 subdomains (SD1 and SD2); Q954H and N969K in the heptapeptide repeat (HR)1; and S1252P and D1260N in HR2 (Fig. 5; Table S2). Moreover, G142D, K147E, W152R, F157L, K187T, I210V, V213G, S221R, and G257S in the NTD, T478K, and S494P in RBD were distinct mutations on 16 March 2023, with a peak viral load. T478R, F490S in RBD, and T583E in SD1/SD2 were distinct mutations on 14 August 2023. In addition, A26S in the NTD and S477N in the RBD were distinct mutations on 16 March and 14 August 2023.

## DISCUSSION

In this study, we demonstrated that the detection and genomic characterization of SARS-CoV-2 lineages present in the wastewater treatment plants of Addis Ababa is a viable alternative to collecting and analyzing clinical samples. We utilized information on mutation frequency in wastewater samples, employing Freyja for the proportions of different SARS-CoV-2 lineages in a sample and our signature mutation analysis. This approach effectively identified lineages circulating at low frequencies in wastewater and helped mitigate biases associated with samples containing a mixture of variants (25).

The SARS-CoV-2 viral load of the target genes in the wastewater sample started to increase on 6 March 2023, with a high percentage of the CH.1.1 variant. During the CH.1.1 peak on 16 March 2023, a distinct set of mutations was observed in the spike protein, including G142D, K147E, W152R, F157L, K187T, I210V, V213G, S221R, and G257S in the NTD and T478K and T494P in the RBD. These mutations are located at sites targeted by neutralizing antibodies and may also influence the spike protein’s binding affinity to its receptor, angiotensin-converting enzyme 2 (26, 27). For instance, the K147E amino acid mutation can substantially reduce the neutralization activity of the sera. The G142D mutation is predicted to reshape the NTD surface topography, including the antibody-binding “super site” formed by the N3 and N5 loops, and, when combined with T95I, is linked to higher viral loads (28). The V213G substitution may contribute to immune evasion by reducing human leukocyte antigen (HLA-DR) binding affinity (29). Moreover, a T478K mutation identified within the critical receptor-binding motif of the functional domain of the S gene is likely linked to the infectivity and pathogenesis of the SARS-CoV-2 variant (30). Furthermore, the S494P mutation exhibits strong and significant resistance to the binding of the spike protein with monoclonal antibodies such as Sotrovimab (31). The fact that specific amino acids were found at sites targeted by neutralizing antibodies indicates the evolutionary selection pressure on SARS-CoV-2 by human immune systems.

Overall, since the peak in March 2023, the XBB.1.5 variant continued to increase in prevalence in April and May 2023, despite decreasing viral load in wastewater up to 11 May 2023. XBB.1.5 appeared to outcompete other variants as the primary circulating strain, possibly reflecting its heightened transmissibility and superior immune-evasion capabilities (32). The period between May and July 2023 saw relatively stable viral levels, indicating that the virus was circulating at a steady rate, but without any major spikes. The situation seemed under control, with no dramatic increases in infections or significant changes in the dynamics of the virus. The sudden rise in sewage SARS-CoV-2 viral load in August 2023 suggests a new wave that could have been caused by various factors, including the emergence of new subvariants, changes in public behavior (e.g., reduced precautions, travel, etc.), and seasonal factors (rainy season). During the peak of the viral load of 14 August 2023, the following spike protein mutations were observed: S477N, T478R, F486L, F490S, and T583E in the RBD. The amino acid T478R mutants may enable the pathogen to escape immune recognition (33); the S477N mutation enhances the pathogen’s ability to escape the immune system by altering its interaction with the MHC-II antigen presentation pathway (34). Finally, the F486L unique mutation could potentially increase the virus’s ability to infect human cells (35), while the F490S mutation might decrease vaccine effectiveness, possibly by allowing the virus to evade antibodies produced through vaccination (36). These mutations demonstrate further increased variability due to selection forces by human immune systems.

During the surveillance period, a representative from the Public Health Emergency Management (PHEM) team was included to ensure timely communication with the medical community regarding any increase in cases. Given that most cases were asymptomatic, the PHEM team leveraged this information to issue early warnings and conduct community-focused educational outreach. Consequently, the response strategy shifted from health facility-based testing to the implementation of mass vaccination campaigns within the community.

A small sample size is one limitation of this study, as it may not fully capture the diversity of SARS-CoV-2 variants present in the population. Another key concern is the generalizability of the findings to the rest of Ethiopia, particularly when comparing urban and rural areas, where population density, healthcare access, and transmission dynamics may differ significantly. However, given the nature of SARS-CoV-2, which tends to spread more efficiently in densely populated areas with higher viral shedding, the likelihood of missing circulating variants in rural settings is relatively low. Nonetheless, additional sampling from diverse locations would strengthen the study’s conclusions.

In conclusion, in the wastewater treatment plants in Addis Ababa, the XBB.1.5 and XBB* variants were the most prevalent circulating lineages, and these results appeared consistent with (limited numbers of) clinical samples derived from individual patients.

In all, we describe in this paper that wastewater is a reasonable alternative for clinical sample-based epidemiological monitoring of SARS-CoV-2, particularly in times of low COVID-19 report presence. We recommend continued alertness by using wastewater screening on a regular basis in key cities in Ethiopia, particularly those that are connected to relatively large numbers of incoming visitors. Sequencing of airplane wastewater samples of Ethiopian Airlines arriving from high-risk countries (also for other pathogens, such as mpox and Ebola) could be considered too.

## MATERIALS AND METHODS

### Sampling and extraction

The study design and sample collection sites were detailed in a previous study (23). Briefly, the sample collection was conducted between March and November 2023 at three wastewater treatment plants in Addis Ababa. A total of 323 influent samples were collected using the Moore swab method (37). Each Moore swab was squeezed of all liquid into a sterile container from which a 10 mL wastewater aliquot was taken using a 15 mL tube for virus capture and extraction. The viral concentration step followed the Ceres technology protocol (38). In total, 150 µL of Nanotrap Microbiome A Particles (SKU#44202, Ceres Nanoscience Inc., Manassas, VA) and 100 µL of Nanotrap Enhancement Reagent 2 (ER2) (SKU# 10112, Ceres Nanoscience Inc., Manassas, VA) were used to trap the virus in a magnet rack. RNA extraction was done using QIAamp Viral RNA Mini Kit (Cat. no. 52906, QIAGEN, Hilden, Germany) following the manufacturer’s instructions (39).

### Quantitative reverse transcriptase polymerase chain reaction

TaqPath COVID-19 (Thermo Fisher Scientific, Waltham, MA) quantitative reverse transcriptase polymerase chain reaction (qRT-PCR) reaction master mix was prepared according to the manufacturer’s instructions (40). Briefly, a total of 15 µL master mix was added to each well of the plate. Ten microliters of extracted nucleic acid was added to the assay wells containing the master mix. In the Plate Setup window of QuantStudio 5 (Thermo Fisher Scientific, Waltham, MA), FAM, VIC, ABY, and JUN dyes were used as reporter dyes for the viral targets of the primers and probes: ORF1ab, Nucleocapsid (N) gene, Spike (S) gene, and MS2 phage control, respectively. Thermal cycling conditions included 2 minutes uracil-N-glycosylase incubation at 25°C, 10 minutes reverse transcription at 53°C, 2 minutes at 95°C for reverse transcription deactivation, and initial activation of Speed Star HS DNA polymerase, followed by 40 cycles of 3 seconds denaturation at 95°C and 30 seconds annealing/extension at 60°C. All samples with cycle threshold (Ct) values of ORF1ab, N gene, and S gene <37; MS2 <32 were considered positive according to the manufacturer.

### Library preparation and sequencing

Ninety-five samples were selected for sequencing based on their Ct values, ensuring that each sample contained at least two target genes with Ct values of 32 or lower. The library preparation and sequencing process followed the Illumina COVIDSeq protocol (Illumina Inc., San Diego, CA, USA) (41), involving cDNA synthesis using random hexamers and reverse transcriptase to amplify isolated RNA for library preparation. A multiplex PCR was then employed to amplify the generated cDNA, utilizing COVIDSeq kit V4 primers to produce 98 amplicons covering the SARS-CoV-2 genome. Following PCR amplification, the product underwent indexing for Illumina MiSeq UD Indexes Set 1 for tagmentation and adaptor ligation, adhering to the manufacturer’s guidelines for further enrichment and cleaning. All samples were processed in batches in a 96-well plate containing either COVIDSeq or nuclease-free water. The pooled sequence library was quantified using a Qubit 4.0 fluorometer with High Sensitivity DNA assay and normalized to a concentration of 4 nM before denaturation with 0.2 N NaOH. Subsequently, the denatured sequence library was loaded onto the flow cell following the MiSeq workflow according to the manufacturer’s instructions at the Integrated Pathogen Genomics and Bioinformatics Facility at EPHI. Dual-indexed paired-end sequencing was conducted with a read length of 350 bp using the MiSeq Reagent Kit v3 with 300 cycles.

### Sequence analysis

#### Quality control checks and variant determination

FASTQ files exported from the MiSeq instrument were uploaded to the Terra.bio platform and trimmed, filtered based on sequence quality, assembled, and mapped to the reference genome (MN908947.3) using Freyja_FASTQ_public health bioinformatics (PHB) workflow (PHB v2.0.1; https://dockstore.org/workflows/github.com/theiagen/public_health_bioinformatics/Freyja_FASTQ_PHB:main?tab=info), which was developed by Josh Levy from the Andersen Lab (https://andersen-lab.com/) and Theiagen Genomics (42, 43). FastQC and Trimmomatic/Fastp were used for the removal of adapters and low-quality sequence data. Fastq-scan was used to read a FASTQ and output summary statistics (read lengths, per-read qualities, and per-base qualities) in JSON format. BBDuk was used for host decontamination (removal of ribosomal RNA) and to combine data-quality-related trimming. The minimum quality score and trimmomatic_min_length were 33 and 25, respectively, to keep during trimming. Metaspade (metaspade-version v3.12.0) was used for *de novo* assembly. QUAST was used for evaluating assemblies both with a reference genome, as well as without a reference. Kraken2 (kraken-version 2.1.2) was used for metagenomic classification of the organism’s genome sequence based on the K-mer principle and removal of reads shorter than 25. Burrow-Wheeler Aligner was also used for mapping the cleaned FASTQ sequence reads to a reference genome (SARS-CoV-2). Samples with at least 2× genome coverage were included in Freyja_Plot_PHB (https://dockstore.org/workflows/github.com/theiagen/public_health_bioinformatics/Freyja_Plot_PHB) analysis and Freyja_Dashboard_PHB (https://dockstore.org/workflows/github.com/theiagen/public_health_bioinformatics/Freyja_Dashboard_PHB:main?tab=info) visualization. To capture the dynamics of virus evolution and spread, we used Freyja_Plot_PHB, a tool to aggregate multiple samples using output from Freyja_FASTQ_PHB to generate a plot that shows a fractional abundance of virus variants or lineages present in wastewater, with the option to plot with sample collection date information. We also used Theiagen Genomics Freyja_Dashboard_PHB workflow in Terra.bio to generate an interactive visualization using the data of Freyja demixed output files generated from the Freyja_FASTQ_PHB workflow.

The variants of concern of SARS-CoV-2 in clinical samples were analyzed using Nextclade (44). Nextclade (https://clades.nextstrain.org) aligns viral genomes to a reference sequence, calculates several quality control metrics, assigns sequences to a clade or variant, and identifies changes in the viral proteins relative to the reference sequence.

#### Amino acid mutation analysis

Since SARS-CoV-2 RNA in wastewater is fragmented, and fragments, hypothetically, originate from multiple individuals (generally infected with genetically distinct lineages), the generation of consensus sequences from wastewater samples is not appropriate because the consensus sequences cannot be interpreted as representing a viral haplotype present in individuals in the population. Therefore, SC2_wastewater_variant_calling (https://dockstore.org/workflows/github.com/CDPHE-bioinformatics/CDPHE-SARS-CoV-2/SC2_wastewater_variant_calling) in Terra.bio and trimmed_sort_bam file (gs://fc-c5033672-b2fb-47ff-9d93-3da0e8b4b053) from the previous output as input was used for amino acid mutational analysis.

### Visualization of amino acid changes using a scatter plot

We used the amino acid variation data file generated by the Terra.bio bioinformatics pipeline. Using the amino acid variations data output file from the Terra.bio bioinformatics pipeline and the generated Microsoft Excel file containing all amino acid variations and their respective read frequencies, a heatmap was generated and interpreted to visually identify patterns of mutation abundance in the spike gene at each date of collection using R (version 4.2.0) software and Stata version 14.

## Data Availability

The SARS-CoV-2 genomes generated in this study have been submitted to the National Library of Medicine, National Center for Biotechnology Information (NCBI) Sequence Read Archive under BioProject ID PRJNA1225690. The reference gene of SARS-CoV-2 was extracted from NCBI, accession number MN908947.3. The dates and PCR results are provided in Table S2. The Ethiopian clinical sample sequence data were abstracted from GISAID with accession numbers EPI_ISL_19469718-19469911 and EPI_ISL_19470219-19470281.

## SUPPLEMENTAL MATERIAL

Table S1 msphere.00229-25-s0001.xlsx Overall wastewater samples with variants among the quality control passed samples.

Table S2 msphere.00229-25-s0002.xls Overall samples with amino acid mutation among wastewater samples having greater than 2× coverage.

ASM does not own the copyrights to Supplemental Material that may be linked to, or accessed through, an article. The authors have granted ASM a non-exclusive, world-wide license to publish the Supplemental Material files. Please contact the corresponding author directly for reuse.
